# The European Union’s Health Technology Assessment Regulation (EU-HTA R) Will Prosper Despite Major Setbacks

**DOI:** 10.3390/jmahp14030045

**Published:** 2026-08-03

**Authors:** Mondher Toumi, Imen Soussi, Bruno Falissard, Steven Simoens, Asma Jouini, Maarten Postma, Juergen Wasem, Oriol Solà-Morales, Laurent Boyer, Claude Dussart, Borislav Borissov, Renato Bernardini, Stefano Capri, Jaime Espin, Pascal Auquier

**Affiliations:** 1 CEReSS/UR3279—Health Services Research and Quality of Life Center, Aix-Marseille University, 13385 Marseille, France; 2 Clever Access, Tunis 1058, Tunisia; 3 CESP, INSERM U1018, Université Paris-Saclay, 94800 Villejuif, France; 4 Department of Pharmaceutical and Pharmacological Sciences, KU Leuven, 3000 Leuven, Belgium; 5 Department of Health Sciences, University Medical Center Groningen, University of Groningen, 9700 AB Groningen, The Netherlands; 6 Faculty for Economics and Business Administration, Institute for Health Care Management and Research, University Duisburg-Essen, 45127 Essen, Germany; juergen.wasem@medman.uni-due.de; 7 Fundació HiTT, Aragó 60, Pral. 1a, 08015 Barcelona, Spain; 8 UR 4129 P2S Parcours Santé Systémique, Université Claude Bernard Lyon 1, 7-11 Rue Guilllaume Paradin, 69008 Lyon, France; 9 Pharmacie Centrale, Hospices Civils de Lyon, 57 Rue Francisque Darcieux, Saint-Genis-Laval, 69230 Lyon, France; 10 Department HTA, Faculty of Public Health, Medical University of Sofia, 1527 Sofia, Bulgaria; borislav.borissov@prescriptia.com; 11 Department of Biomedical and Biotechnological Sciences (BIOMETEC), Section of Pharmacology, University of Catania, 95124 Catania, Italy; 12 School of Economics and Management, Cattaneo-LIUC University, 21053 Castellanza, Italy; 13 Andalusian School of Public Health/Escuela Andaluza de Salud Pública (EASP), 18011 Granada, Spain; 14 CIBER en Epidemiología y Salud Pública (CIBERESP)/CIBER of Epidemiology and Public Health (CIBERESP), 28029 Madrid, Spain; 15 Instituto de Investigación Biosanitaria ibs.GRANADA, 18012 Granada, Spain

**Keywords:** EU-HTA, semantic HTA EU-HTA regulation, words signification, disguised

## Abstract

**Background:** The EU Health Technology Assessment Regulation (EU-HTA R), effective January 2025, mandates Joint Clinical Assessments (JCAs) to harmonize HTA across Member States. However, its implementation raises fundamental questions about methodological coherence, institutional capacity, and epistemological alignment. **Objectives:** This manuscript (1) systematically assesses whether the stated strategic and operational objectives of the EU-HTA R are achievable under current implementation conditions; (2) examines the implications for EU institutional legitimacy if these objectives are not met; and (3) proposes an epistemological framework as a prerequisite for developing a coherent joint HTA methodology. **Methods:** We conducted a critical policy analysis of the EU-HTA R, its implementing guidance documents, and published templates, supplemented by a comparative review of Member State HTA methodologies and their underlying philosophical foundations. **Results:** The analysis reveals that the EU-HTA R is unlikely to achieve its strategic goals under current conditions. Key findings include: guidance documents of substandard methodological quality; a restricted assessment scope that excludes scientific judgement and contextualization; insufficient resources and additional workload for national HTA bodies without reducing existing obligations; unresolved epistemological divergences among Member States spanning Bayesian vs. frequentist approaches, Fisher vs. Neyman–Pearson frameworks, and utilitarian vs. deontological ethical foundations; and procedural shortcomings in stakeholder consultation and expert involvement. These shortcomings risk undermining the epistemic authority and legitimacy of EU institutions. **Conclusions:** Prior epistemological and normative alignment across Member States is a prerequisite for any robust shared HTA methodology. Revisions to the EU-HTA R and comprehensive updates of guidance documents are necessary, with concrete safeguards—including independent peer review, identified authorship, and adequate resourcing—to ensure substantive rather than merely nominal implementation. A phased roadmap is proposed: establishing clear objectives, aligning epistemological foundations, developing institutional structures, and creating operationally consistent guidance.

## 1. Introduction

The European Parliament, in its resolution of 2 March 2017 on European Union (EU) options for improving access to medicines, called on the Commission to propose legislation on a European system for health technology assessment (HTA) to harmonize transparent HTA criteria to assess the added therapeutic value and relative effectiveness of health technologies compared with the best available alternative [1].

In December 2021, the EU-HTA R, formally known as ‘Regulation (EU) 2021/2282 of the European Parliament and of the Council of 15 December 2021’ [2], on HTA and amending Directive 2011/24/EU [3] was adopted. It entered into force with implementation starting in January 2022 for oncology products and advanced therapeutic medicinal products (ATMPs) in January 2028 for orphan designated products and in January 2030 for all products.

While the EU-HTA R stated several objectives, it is important to analyze the feasibility of achieving these objectives as no impact study was conducted before this law was published. An impact analysis study was performed by the EU Commission’s internal staff, not the EU Parliament, after the consultation on the EU-HTA model [4]. The aim of this opinion manuscript is to assess whether the stated objectives in the EU-HTA R are achievable, discuss the implications of the potential level of achievement of these objectives, and propose an epistemological framework that aligns the HTA fundamentals with the objectives of a joint EU-HTA analysis framework.

The central thesis of this manuscript is that the EU-HTA R’s objectives are unlikely to be fully achieved under current implementation conditions, primarily because the regulation and its guidance documents were developed without prior alignment on the epistemological foundations necessary for a coherent joint assessment framework. This claim is supported through three complementary lines of analysis. Part I systematically evaluates the stated strategic and operational objectives of the EU-HTA R against the published guidance documents, resource constraints, and structural barriers, demonstrating a significant gap between legislative ambition and implementation capacity. The Discussion and Conclusions section examines the broader institutional consequences: if the EU-HTA R fails to deliver scientifically credible assessments, this risks undermining not only the regulation itself but also the epistemic authority and legitimacy of European institutions in the domain of evidence-based governance. Part III identifies the root cause of these implementation difficulties—unresolved epistemological divergences among Member States—and proposes a structured framework for achieving the philosophical alignment that is a prerequisite for any robust shared methodology.

An important distinction must be drawn between critiquing the regulation itself and critiquing its implementation. Our analysis addresses both dimensions. At the regulatory design level, the EU-HTA R sets potentially unachievable strategic objectives given structural constraints inherent to the European context—particularly subsidiarity in healthcare, deep heterogeneity among Member State HTA traditions, and the exclusion of scientific judgement from the assessment scope. These are not implementation failures but architectural limitations embedded in the legislative text itself. At the implementation level, the guidance documents and templates developed under the regulation exhibit deficiencies—lack of systematic literature review, absent authorship, and inadequate stakeholder consultation—that represent failures of the process rather than of the legislative mandate. This dual critique is essential for identifying appropriate corrective actions: regulatory design flaws require legislative revision, while implementation shortcomings may be addressed through improved governance and methodological rigour within the existing framework.

A note on the evidence base is warranted. The EU-HTA R entered into force in January 2025, with guidance documents finalized only in late 2024. Peer-reviewed literature on its implementation is therefore necessarily limited at this stage. Our analysis draws on the regulatory text itself, official EU documents, published stakeholder consultations, and the emerging academic literature where available. Certain references include policy briefs and position papers from organizations directly involved in the implementation process; these are cited not as impartial evidence but as primary sources documenting stakeholder perspectives and institutional responses. Where preprints have since been published in peer-reviewed journals, we cite the published versions.

## 2. Part I: Will EU-HTA Regulation Objectives Be Achieved?

In this section, the objectives are categorized into three segments: 1. strategic objectives, 2. operational objectives as outlined in the EU-HTA R, and 3. specific objectives as described in the regulation, primarily within the recitals and detailed provisions. To prevent redundancy due to overlapping objectives, each is addressed only once and cross-referenced as needed. Following the listing of the objectives, their likelihood of being achieved is evaluated.

### 2.1. Strategic Objectives

The Regulation (EU) 2021/2282 explicitly outlines two overarching strategic objectives:

- “High level of health protection for patients and users: The law aims to ensure a “high level of protection of health for patients and users” through better assessment of health technologies”.
- “Smooth functioning of the internal market for health technologies: It simultaneously seeks to guarantee the “smooth functioning of the internal market” with regard to medicinal products, medical devices and in vitro diagnostic devices”.

“These two broad goals are pursued simultaneously and are considered inseparable and equal in priority”.

### 2.2. Operational Objectives

The EU-HTA R aims to accomplish the following operational and core objectives:

- To improve the availability for EU patients of innovative technologies in the area of health: The regulation aims to accelerate patient access to innovative and effective health technologies. Covered under the first strategic objective: high level of health protection for patients and users.
- To harmonize HTA criteria and increase transparency, to promote convergence in HTA tools, procedures and methodologies: A core objective of the regulation is to harmonize HTA processes among Member States (MSs) to remove divergences in clinical assessments, fostering EU-wide cooperation and alignment on methods and principles.
- To ensure efficient use of resources and strengthen the quality of HTA across the EU: By establishing joint clinical assessments (JCAs) and consultations, the regulation reduces duplication of efforts, pools expertise, and improves the scientific quality and sustainability of HTA cooperation.
- To improve business predictability for health technology developers (HTDs): The regulation introduces a unified EU-level submission process and clearer, streamlined procedures, which reduce administrative burdens and increase predictability for developers regarding evidence requirements and timelines.

### 2.3. Specific Objectives

Beyond the above, the regulation pursues more specific objectives throughout its text (in the recitals and detailed provisions), even if not formally labelled as objectives in the law. Each of the below points represents an objective or an intended effect that the HTA regulation aims to achieve, as evidenced by the text of the law. These specific objectives collectively support the regulation’s overarching strategic goals. These objectives are sometimes overlapping with the operational objectives. These include:

- To comply with the state of the art of medical sciences or evidence-based medicine, ensuring quality, transparency, and independence: Ensuring that joint HTA work is conducted with the highest scientific quality, transparency, and independence. The regulation states that joint work should “aim to achieve the highest level of quality, transparency and independence.”
- Timely and up-to-date assessments: Requiring that JCA reports be completed in a timely manner and kept up-to-date as new evidence emerges.
- Inclusive stakeholder and expert involvement: Promoting inclusive and transparent HTA processes by involving a broad range of stakeholders and experts.
- To comply with the state of the art of medical sciences or evidence-based medicine, ensure the highest quality reference is applied.
- To adhere to the highest standards of quality, impartiality, transparency, and independence.
- To minimize duplicate efforts for MSs and HTDs, avoid presenting the same evidence to multiple MS HTA bodies and reduce the HTD workload for MS submissions.
- To adhere to the highest administrative standard, ensure the highest EU administrative standards apply to this joint MS initiative supported by the EU, while not an EU agency or output.
- To adapt to the specific needs of emerging health technologies, such as vaccines, orphan drugs and ATMPs, where data may not be readily available.
- To show flexibility by focusing primarily on randomized clinical trials (RCTs) but considering alternative evidence such as observational studies.
- To reduce the cost of HTA submissions for HTDs, by reducing the volume of work for multiple submissions as MS will align on the JCA report, this would reduce the workload and associated internal and external budget for HTDs.

### 2.4. Assessment of Achievement of Objectives

This opinion evaluates whether the regulation aligns with its strategic, operational and specific goals based on the current situation. The implications of the findings are discussed within a broader policy perspective. This analysis focuses on medicinal products only.

As an opinion manuscript, the conclusions presented here necessarily involve expert judgement. However, to ensure transparency, we make the ground for that judgement explicit. Our assessments of whether individual objectives are “likely” or “unlikely” to be achieved rest on five criteria: (1) potential gap of each stated objective with the content, scope, and prescriptive quality of the published guidance documents and templates; (2) analysis of implementation timelines and resource constraints as reported by Member States and documented in official and unofficial communications; (3) review of published critiques, including peer-reviewed analyses and formal stakeholder feedback submitted during consultation processes; (4) assessment of structural and institutional barriers identified in the broader HTA and health policy literature; and (5) consideration of the substantial ongoing debate in scientific forums and learned societies regarding the regulation’s methodological foundations. Where our conclusions go beyond what these sources directly establish, we identify this as our own interpretive assessment.

We review and discuss step by step:

- The improvement of patients’ health protection
- The smoothing of the single market for medicinal products
- The achievement of operational and specific objectives

To streamline the assessment, redundant objectives are consolidated and addressed concurrently.

In addition, to provide a standardized assessment, we applied the OECD DAC Evaluation Framework to the EU Health Technology Assessment Regulation. The framework and its results are presented in the Supplementary Materials. It was applied by five co-authors with expertise in health policy evaluation, including the use of evaluation frameworks, and in public health, who reached consensus through a deliberative process. This standardized assessment was consistent with the co-authors’ judgement and supported the results.

#### 2.4.1. Improvement of Patients’ Health Protection

In the EU, there is a noticeable variation in access to medicinal products among MSs. The time to access and availability of innovative medicinal products differ significantly across MSs, as widely reported in the literature [5,6,7,8,9]. Although HTA policies vary considerably and may account for differences in rare instances, several studies have shown that HTA decisions in France and Germany tend to be relatively similar [10,11,12]. Despite the different policies between these two countries, orphan designated drugs (ODD), for example, achieve a comparable level of patient access [13]. The same findings have been published for Scotland and England [14,15,16]. While other authors confirmed the similarity of decisions between Italy, France and Germany [17]. So HTA does not appear to be the source of disparity in access.

The main reason for disparities in patient access is affordability differences and the legal framework. For instance, in Germany, reimbursement is mandated by law for all products covered under health insurance according to the German Social Code Book 5 (SGB V) [18].

There is a close linear relationship between Gross Domestic Product (GDP) per inhabitant and the proportion of newly approved medicines available. When adjusted for GDP per inhabitant, the price of orphan drugs appears significantly higher, ranging from one to ten times more expensive, in EU countries with lower GDP per inhabitant [19]. This raises an important equity concern. Research indicates that affordability is a key factor affecting patient access to innovative therapy in the EU [20]. As a result, it is unlikely that the EU-HTA-R will change the current disparity in patient access to innovation across EU MSs, unless some states subsidize others. This will require EU procurement and a differential contribution between MSs to align price and affordability. While a report to the EU Commission on a single EU price concluded it would not be feasible [21], following the procurement of COVID-19 vaccines, the EU Commission is considering the establishment of a legal framework—through the Critical Medicines Act—to facilitate EU-level collaborative procurement especially for critical medicines and medicinal products of common interest [22]. This may serve as a basis for expanding the scope of EU procurement to align access across the EU.

However, some researchers suggest that the EU-HTA R may slow down national reimbursement processes for medicines, delaying market access. This regulation misses the chance to expedite medicine reimbursements [23].

#### 2.4.2. Smoothing of Single Market for Medicinal Products

Enhancing the functionality and effectiveness of the single EU market aims to ease the free movement of goods, services, capital, and people among MSs.

The single EU market for medicinal products encounters challenges due to a variety of regulations at national and regional levels within MSs, primarily influenced by subsidiarity in healthcare [24].

Subsidiarity in healthcare means that health policy decisions should be made at the local level, usually by individual MSs. This results in significant policy differences between MSs, making the single market inefficient and dysfunctional [25].

Several factors contribute to this diversity, as demonstrated by the following non-exhaustive list of varied policies:

- The Beveridge and Bismarck models are different approaches adopted by various healthcare systems. These models result in differing perspectives on patient coverage, efficiency, equity, and out-of-pocket payments for patients, etc. [26,27].
- National regulatory frameworks include HTA policies, distribution policies, pharmacovigilance requirements, prescription conditions, and advice to HTDs from both central and national authorities.
- Lack of unified pricing and reimbursement policies, with negotiations and documentation often needing to be in local languages.
- Economic differences exist between large and small markets, affecting attractiveness and eventually affordability.
- Packaging, labelling requirements, and multiple languages in the EU complicate drug circulation across MSs.
- Supply chain complexity and anti-counterfeiting measures impact logistics.
- Parallel trade discourages or delays launches in low-price markets.
- Higher shortage of medicinal products in smaller and lower-cost markets.
- Heavy reliance on external suppliers for innovative therapies and APIs for generics and biosimilars is worsening shortages unevenly across the EU.

##### EU Legislative and Policy Responses

This list of dysfunctions and inefficiencies in the EU single medicinal products market shows the effort needed for a functional single market. The EU Commission is working on this challenge but has seen limited success so far. On 26 April 2023, the European Commission proposed a new package of pharmaceutical legislation aimed at revising many of the currently applicable regulations [28]. The explicit purpose of this proposal is to create a true single market for medicinal products and improve the health of the EU population.

More recently, the Critical Medicine Act (CMA) marked a significant effort by the EU to tackle drug shortages and supply-chain issues [22]. It aims to ensure access to critical medicines through prioritizing EU manufacturing, joint procurement, and strategic partnerships. The CMA seeks to enable collaborative, equitable procurement by MSs and the Commission, with the objective of enhancing purchasing power and ensuring supply security [29]. Success will require addressing industry concerns about joint procurement and equitable implementation across MSs [30]. Broadening the scope of CMA may expand the products concerned by EU procurement. A report to the EU Commission did not support the feasibility of a single EU price [21]. The concept of a single price for medicinal products across the EU remains deeply controversial due to conflicting national priorities, market fragmentation, and debates over affordability versus innovation [21,31,32].

In theory the harmonization of EU-HTA R may contribute to the functioning of the single EU market for medicinal products, but it is unlikely to materialize. This question is at the top of the EU Commission’s agenda.

##### Implications for the EU-HTA Regulation

EU-HTA R focuses primarily on clinical analysis and does not include several other aspects as described in the EUnetHTA core model [2]. Aspects such as cost-effectiveness analysis may be considered central to HTA decisions in some countries leaving clinical assessment less critical. Even among HTA bodies driven by clinical evidence, major differences remain. In Germany, the Institute for Quality and Efficiency in Health Care (IQWIG) assesses clinical effect size using the upper limit of the 95% confidence interval for efficacy rate ratios. In France, the Haute Autorité de Santé (HAS), particularly the Commission de la Transparence considers the absolute difference in efficacy outcomes. Each country will keep its own decision analysis framework despite EU-HTA R.

EU-HTA R evaluates only the basic elements (clinical evidence) without context or judgement, leaving appraisal out of the process. Thus, within the current context, the EU-HTA R is unlikely to affect the single EU market’s efficiency or functioning to any magnitude. However, the EU and MSs are engaged in several initiatives to improve the functioning of the single EU market for medicinal products.

The analysis of the regulatory text and implementation context presented above suggests that the strategic objectives of the EU-HTA R face substantial barriers to achievement. This assessment emerges from comparing the scope of the legislative ambitions with the operational constraints documented in the preceding sections—including the restricted assessment scope, resource limitations, and unresolved methodological divergences among Member States. Given the gap between stated objectives and implementation capacity, it is important to consider the reasons behind the issuance of this policy by the European Parliament and the Commission.

EU regulations must set achievable strategic objectives; otherwise, the validity of the regulation is in question unless the strategic objectives are revised. This is an existential question for this regulation.

#### 2.4.3. Achieving Operational and Specific Objectives

The operational objectives may face challenges in being achieved under the current regulations, methodological guidance, and templates available. Three potential concepts for the operational objectives include: (i) aligning with the state of the art in medical sciences, (ii) adhering to the highest administrative standards of the EU, and (iii) avoiding duplication of effort. They are unlikely to be achieved.

##### To Harmonize HTA Criteria with Transparency

The scope of the EU-HTA R is limited, ignoring all non-clinical aspects of HTA and leaving decisions like appraisal to individual MSs as outlined in the single EU market section of this manuscript. Therefore, HTA in the EU will mostly remain under MS’ control, with varying methodologies across them. Despite some cross-border initiatives like the Beneluxa initiative [33], joint HTA and procurement decisions are rare. With divergences outweighing convergences, the EU-HTA R will hardly contribute to the harmonization of HTA criteria or transparency. While guidance documents are supposed to provide clear recommendations to assess clinical evidence in practice, they remain vague and subject to interpretation of MS for several topics like validity, multiplicity, statistical precision or indirect treatment comparisons [34,35,36,37]. Therefore, heterogeneous interpretation of guidance documents at the assessor and MS HTA level is expected. Finally HTA MSs are expected to give due consideration to the JCA report, but due consideration is not defined and is left to HTA MSs’ appreciation. There are no obligations. Therefore, it is unlikely that HTA criteria will be harmonized and transparency will increase. Sharing best practices may contribute to unifying understanding and interpretation of clinical evidence to enhance harmonization.

JCA regulation standardizes the epistemological process using the Population, Intervention, Comparison, and Outcome (PICO) framework. Though less flexible, this allows Member States to adapt it to their specific needs, especially useful for countries with emerging HTA. Indeed, smaller Member States and those with nascent or resource-constrained HTA systems stand to benefit substantially from well-conducted JCA reports, which could provide a rigorous evidence base that these countries would otherwise lack the capacity to produce independently. This potential benefit reinforces rather than diminishes the importance of ensuring high methodological quality in JCA outputs: if the assessments that smaller Member States rely upon are themselves of substandard quality, the promised benefit becomes illusory.

##### To Improve the Availability for EU Patients of Innovative Technologies

This topic has been addressed under the first strategic objective.

##### To Ensure Efficient Use of Resources and Strengthen the Quality of HTA

This section has been divided into two topics: the first “ensure efficient use of resources” is addressed within the section “minimize duplication of effort for MS and HTD” and the second “strengthen the quality of HTA” is addressed in the section “Comply with the state of the art of medical sciences or evidence-based medicine (EBM)”.

##### To Improve Business Predictability for HTDs

The EU-HTA R introduces significant uncertainty for HTDs rather than improving predictability. The 90-day timeframe for submissions is too short, requiring HTDs to prepare in advance without knowing the exact scope, leading to high uncertainty if the anticipated scope differs from reality. There is no applicant documentation specifying methodological requirements for HTDs. The lack of clarity and low quality of assessors’ guidance documents make it unclear which methodology to follow. The interpretation of the JCA report “due consideration” by different Member States and its actual impact are unknown. Additionally, the restrictive scope of evidence acknowledged by JCA guidance offers little hope for a positive assessment. The EU-HTA R is unlikely to improve predictability for HTDs and may instead worsen it.

##### To Comply with the State of the Art of Medical Sciences or Evidence-Based Medicine (EBM)

The EU-HTA R guidance often references EBM but does not follow its principles. The guidance development was not preceded by a systematic literature review, lacks authorship, and lacks external review. These guidance documents only represent expert opinion at best, which constitutes the lowest level of evidence in EBM. It is unexpected that the guidance meant to assess uncertainty carries so much uncertainty itself by ignoring EBM principles. Aligning with the state of the art in medical sciences requires fully revisiting how the guidance is developed. The established methodology and critical steps for developing guidelines should be followed. Genuine public consultation and the engagement of qualified experts and HTDs are essential. This approach should substantially improve the situation. The current level of expertise allocated to the development of these guidance documents appears insufficient, resulting in misconceptions and several errors.

This approach is consistent with what Isaacs and Fitzgerald [38] critically termed “Eminence-Based Medicine”—a practice in which recommendations are formulated by designated experts relying primarily on their individual judgement and professional authority rather than on systematically derived evidence. Applied to the EU-HTA context, guidance documents developed without a systematic literature review, without identified authorship, and without external peer review risk reproducing this pattern. Such an approach is unlikely to strengthen the quality and credibility of HTA.

##### To Adhere to the Highest Standards of Quality, Impartiality, Transparency, and Independence

The guidance tends to refer to minimal quality standards based on experts’ opinions instead of a systematic literature review [39]. It often makes arbitrary decisions in recommending tools like ROBINS-I (Risk of Bias in Non-randomized Studies of Interventions) [36] and ICEMAN (Instrument to assess the Credibility of Effect Modification Analyses), or methodologies such as narrative assessment for external validity [40], or shifting the null hypothesis for population-adjusted indirect comparison (PAIC) [35,40,41]. While RCTs are generally preferred, other evidence types like observational studies, single-arm trials and anchored or unanchored indirect treatment comparisons are subject to negative a priori and are not handled impartially. The methodological guidance is aimed at (co-) assessors and does not directly address HTDs’ methodological requirements. Consequently, HTDs must infer from the guidance how a high-quality submission should appear. Ultimately, the guidance documents reflect the viewpoint of specific HTA organizations, lacking a more comprehensive and balanced perspective.

##### Timely and Up-to-Date Assessments

It remains to be seen if the timelines proposed within the regulation and implementing act are feasible for both HTDs and assessors. Already the time for developing submission has been integrated with the regulatory stop of the clock at 120 days. It is considered that this will be 90 days [42,43]. Typically, 50% of products have a stop of the clock of less than 90 days. Janssen, a Johnson & Johnson company, communicated a stop of the clock of only 31 days (1 month) in several cases [44]. The accelerated process follows EMA timelines. While it makes sense for regulatory purposes to shorten timelines, as quality, toxicology, pharmacology, and phase I studies may not be reviewed, the HTA’s clinical evaluation for an indication extension is just as demanding as the initial application.

If the anticipated indication or population is adjusted during the regulatory process, it may or may not affect the PICO. According to a recent webinar by selected JCA subgroup representatives, if there are no PICO changes, the JCA report may be delayed by 180 days. With PICO changes, the delay will be around 330 days [42]. In either case, the JCA report will be available well after marketing authorization. However, more clarity is expected from the JCA subgroup.

The assessment timeframe for the JCA report development and submission is very limited, despite requiring extensive evidence from HTDs that needs to be reviewed. It is uncertain how well HTDs will meet the submission requirements. There are reservations about the ability of JCA assessors to conduct a thorough evaluation within such a tight schedule.

##### To Adhere to the Highest Administrative Standard

It should be feasible to ensure quality, impartiality, transparency, independence, and accountability. This would require that EU procedures be applied rigorously. The EU is a mature institution with numerous laws, case law, rules, and processes designed to secure adherence to high administrative standards. The current situation is characterized by substantial uncertainty about how the process will be and question marks on the guidance documents may arise from the specific structure of the EU-HTA. Unlike an EU agency, the EU-HTA is a group of MSs operating on a voluntary basis, coordinated by the EU, which provides a secretary, infrastructure, and financial resources to achieve a common goal. This specific situation may explain the insufficient adherence to EU administrative standards in the overall EU-HTA process. All guidance documents specify on the front page that this is not an EU Commission document and does not engage the EU Commission. Such an objective is expected to be achieved with time and experience.

##### To Adapt to the Specific Needs of Emerging Health Technologies, Such as Vaccines, Orphan Drugs and ATMPs, Where Data May Not Be Readily Available

The documents (processes, guidance, templates, etc.) show no adaptations to emerging technologies, contrary to what is outlined in the EU-HTA R. As such, they may not be fit for purpose within the context of the regulation’s objectives. Additionally, there is no evident effort to ensure compliance with the requirements established by the EU-HTA R.

##### To Focus Primarily on RCTs but Consider Alternative Evidence Such as Observational Studies

While alternative designs to RCTs are recognized in the various methodological guidance documents, they hold little weight and are not expected to provide any certainty in assessing comparative effectiveness. This discourages HTDs from filing such evidence for JCA. While we acknowledge reservations about these study designs, our focus here is on whether the regulation’s stated objectives are likely to be achieved. We conclude that this objective—genuine consideration of alternative evidence—is unlikely to be met under current guidance.

##### To Minimize Duplicate Efforts for MSs and HTDs

The guidance aims to reduce duplicated work but overlaps in methodological guidance and reporting requirements raise concerns about unusually high levels of required effort.

Furthermore, as the JCA is completely decontextualized and free from judgement, it will provide limited information for evaluating clinical evidence from an HTA standpoint. Beyond the JCA report, a comprehensive critical review of the source clinical evidence on top of the JCA report will be necessary to understand the context and make the appropriate judgements for national appraisal purposes. The JCA report will not prevent a comprehensive review of the whole clinical evidence submitted to integrate the context and judgement while reading.

The structure of the JCA report does not allow reading all information related to a single PICO in a single flow. Information for a single PICO is fragmented and intricately connected in multiple PICOs. There are so far no opportunities to append each PICO as a single report in the Supplementary Materials to ease the reading flow of individual MS-specific PICOs.

Each MS HTA body will have increased workloads, needing to review the clinical evidence sources, the JCA report, integrate this into their final decision, and document how they did it. MS HTA bodies used to receive a comprehensive single submission. Now, they will request country-specific information in a separate document from the JCA submission, disrupting the reading flow and making national assessments less efficient. Specifically, Sweden estimated the need for 17 more staff members to handle these processes, while Italy’s AIFA expressed concerns about resource availability for implementing the new requirements [45]. This approach increases effort rather than reducing it.

For MS HTA bodies, the EU-HTA R creates additional obligations—including JCA participation, report review, and documentation of “due consideration”—without eliminating any existing national HTA requirements. Empirical evidence confirms the magnitude of this additional burden: Sweden has estimated that implementation will require 17 additional staff members, while AIFA (Italy) has raised concerns about the availability of resources needed to meet these new requirements [45]. These new duties compound the workload of national HTA agencies, which must now integrate multiple sources of information from multiple sections addressing multiple PICOs, increasing both the volume of work and the potential for errors. Moreover, the fundamental differences in HTA methodology between MSs will remain unchanged.

Avoiding duplication of effort is complex. There are two perspectives on the issue. One view is that joint HTA practice is inherently beneficial, implying that achieving this benefit justifies the cost. This perspective calls for defining and rationalizing the inherent benefit. Another approach is to consider avoiding duplicating efforts by implementing a joint HTA as a key objective. This would require significantly adjusting the scope of the JCA to include contextualization and full scientific judgement. This shift would make the exercise a comprehensive scientific one, addressing both ontic and epistemic components of the validity of experiments submitted such as direct and indirect evidence. Consequently, the MS would still lead the societal value translation from evidence, via appraisal, reducing the workload of MS HTA bodies as they would receive a thorough assessment, similar to the European Medicines Agency (EMA) assessment.

MSs prefer to retain control over HTA recommendations, especially regarding the impact on drug pricing, which can be a sensitive issue. The JCA will examine multiple PICOs (population, intervention, comparator, and outcomes), up to 13 in recent simulations [46,47], with possible evidence for each PICO varying [48]. A comprehensive scientific evaluation grounded in factual evidence and scientific reasoning will yield distinct assessment outcomes for each PICO, addressing the needs of various groups of countries. By requesting additional evidence, applying distinct HTA methodologies, and conducting appraisals, national HTAs will retain their influence. Therefore, if avoiding duplication of effort is one of the primary operational goals, expanding the scope of scientific assessments to scientific judgement, scientific context, position in the treatment algorithm, or additional clinical evidence that may be requested by MSs, for example could be an option that aligns with MSs maintaining control over the final HTA recommendation and also enhances the quality of JCA deliverables.

##### Inclusive Stakeholder and Expert Involvement

The stakeholder network’s role in JCA submission and report reviews is unclear. HTDs are not involved in HTD scoping, nor consulted or allowed to contribute evidence or challenge consolidated PICOs. This is not inclusive. Experts, clinicians, and patient representatives face uncertainty about their impact. The judgement-free and decontextualized process likely means their input will not be considered. The JCA subgroup and assessors, along with co-assessors, will concentrate exclusively on the ontic component of the experiment, adhering strictly to their guidance documents and guidelines. Assessors will determine if the HTD submission aligns with the guidance and evaluate its impact on the certainty of relative effectiveness. This limits the influence of patients or experts by design. Consulting third parties with minimal impact opportunities remains limited in inclusivity.

##### To Reduce the Cost of HTA Submissions for HTDs

Due to tight timelines (at best 100 days), HTDs face challenges in developing JCA submissions with many uncertainties, adding risk and burden. Using external vendors for predicting and consolidating PICOs at the MSs and EU level will be costly and unpredictable. For single-arm trials, following clinical validity guidance means negative outcomes are known before the JCA report; hence, there is a suggestion to exempt these products from JCA submission. HTDs must continue to comply with national HTA regulations, meeting various methodological requirements, including specific clinical evidence. Overall, EU-HTA R will likely increase the cost, risk, and possibly time for MSs issuing HTA recommendations.

Currently, it is difficult to reframe these strategic objectives. A thorough impact study and extensive consultations with the public, experts and HTDs are necessary. Although the consultations were extensive, they may not have been sufficiently focused or pragmatic. Additionally, limited resources at the EU Commission might have restricted a comprehensive review of the results from the public consultation.

##### To Establish Sustainable EU Cooperation

EU regulation is a binding legislative act that applies directly to all MSs upon entering into force. It becomes part of national law and can be enforced through national courts from that time.

The EU-HTA R came into effect on 12 January 2025. As a binding legislative act, it is directly applicable across all EU MSs uniformly, superseding national laws. Consequently, MSs are obligated to adhere to and collaborate under this regulation.

Experience with the European Medicines Agency shows that once the process is well established, cooperation tends to be proactive and fruitful. It is likely that MSs will develop strong HTA cooperation over time. Establishing sustainable cooperation serves as a means to achieve an objective rather than being an objective itself.

## 3. Part II, Discussion and Conclusions

Although the EU-HTA is not an official EU agency, it creates the impression that it is. The process is governed by the EU-HTA R, with the MS HTA Coordination Group referred to as HTACG, and the EU secretariat handling communications and validations. Any failure to meet objectives will be perceived as an EU failure. Therefore, it is crucial to analyze the potential impact on EU institutions if the EU-HTA R objectives are not met. This section integrates this discussion with our concluding observations, as the implications for EU legitimacy and the broader conclusions are closely interrelated.

### 3.1. The EU-HTA Process Impact on EU Legitimacy

Flawed guidance and inconsistent regulation may undermine the EU’s cross-border efforts. The EU Commission significantly influences the MS Coordination Group on HTA (MS-HTA-CG), despite it not being an agency, by adopting delegated acts and implementing measures with input from a committee representing all EU countries [2].

The MS-HTA-CG might be regarded by the public as an EU agency, despite this not being the case. Europe is experiencing an “agencification” by delegating its power and authority increasingly to agencies. These agencies are not elected by citizens and often gain authority through an arbitrary decision; hence, they are called non-majoritarian institutions. While these agencies emerge from a political and economic decision, unlike political authority derived from elections, these agencies derive their legitimacy through scientific expertise, data analysis, rigorous methodologies, and sound scientific judgement. Their authority is called epistemic authority and is not a new concept [49]. Compliance with such agencies and the voluntary delegation of decision-making authorities are driven by their epistemic authority [50] and value-based/behavioural legitimacy [51]. However, the low quality of methodological guidance documents and the agency’s inability to achieve its strategic and operational objectives will result in a loss of epistemic authority and subsequently a loss of legitimacy. This contributes to the broader issue of diminishing credibility within the EU. The perception of diminished legitimacy of EU agencies is a factor contributing to the rise in sovereign movements within the EU.

### 3.2. Why EU-HTA Will Prosper Despite Major Setbacks

Over the years, EU prerogatives have significantly increased. The integration of MSs’ decision-making power into EU centralized structures is growing fast and is ubiquitous [52,53,54,55,56,57]. The subsidiarity of health care did not prevent the establishment of multiple agencies and initiatives such as the EMA, the European Centre for Disease Prevention and Control (ECDC), the Health Emergency Preparedness and Response Authority (HERA), the European Food Safety Authority (EFSA), the centralized procurement of vaccine medical devices during COVID-19, and the cross-border health care and patients’ mobility regulations.

European leaders are keen on expanding EU integration, regulating more aspects of MS policies. Some even advocate for a federal EU. It is unlikely that EU technocrats, elites, lawmakers, and the Commission will admit the failure of the EU-HTA experience and halt this initiative. In 2028, an assessment of the first three years of the EU-HTA activity will be conducted. Despite setbacks, the project is expected to continue or even expand and to be amended to eventually address several of the reported limitations.

In Mario Draghi’s report, *The Future of European Competitiveness*, September 2024 [58]: *“The HTA Regulation has potential to improve efficiency in the uptake of pharmaceuticals by health systems following their marketing authorization. Considerable resources will need to be made available to achieve this objective. In particular, sufficient expert staff from national HTA bodies and Commission services as well as commensurate funding at EU level for HTA bodies should be freed up to ensure the successful implementation of JCA. These assessments will start as of January 2025 for medicinal products with new active substances for the treatment of cancer and for advanced therapy medicinal products. Consideration could be given to models that allow for the cost recovery of EU-level HTA activities through industry fees. This could include establishing a dedicated structure, following the example of HTA agencies at national level that are fee-charging.”*

This proposal is in line with our article. Lack of funding, resources, expertise and restricted scope may prevent the success of EU-HTA R.

### 3.3. Concluding Remarks

The EU-HTA R may not fully accomplish its strategic goals as outlined in the law. However, some operational goals might be met by revising the guidance documents and templates. But this may not be a relevant purpose for this regulation.

Among the avenues that merit discussion—though it remains highly controversial—is the concept of a unified European list price combined with confidential differential pricing mechanisms. Proponents argue that such an approach could represent an important step towards achieving equitable access and improving the functionality of the single EU medicinal products market. Critics, however, contend that a unified list price could disadvantage lower-income Member States, undermine existing confidential discount arrangements, reduce manufacturers’ willingness to negotiate, and ultimately delay patient access in some markets. The feasibility of such a model also raises complex legal and political questions related to Member State sovereignty over pricing decisions. We present this not as a definitive recommendation but as one avenue for further policy discussion, recognizing that the current EU-HTA R—restricted to assessment of clinical evidence, decontextualized and judgement-free—will not by itself solve the issue of equitable access across the EU. The EU Commission’s joint procurement initiative aims to tackle shortages and may also partially address access inequity among Member States.

The substandard quality of existing guidance documents may compromise the EU-HTA process, affecting the credibility of EU agencies and the European Commission even though EU-HTA is not an EU agency. It is essential to clearly align the strategic and operational objectives of the EU-HTA R.

Revisions to the EU-HTA R and comprehensive updates of guidance documents are necessary. However, it is essential to distinguish between nominal compliance and substantive implementation. One might argue that the existing process already formally addressed many recommended steps—such as stakeholder consultation and methodological review—yet the quality of the resulting guidance documents suggests these steps were insufficient in practice. To avoid repeating this pattern, concrete safeguards should be embedded in the revision process: (1) systematic literature reviews conducted by independent methodological experts as the foundation for guidance development; (2) genuine incorporation of stakeholder feedback with documented, publicly available responses to each substantive comment; (3) independent external peer review of draft guidance documents before adoption, conducted by reviewers with no institutional conflict of interest; (4) clear accountability through identified authorship of guidance documents, ensuring that named individuals bear professional responsibility for methodological quality; and (5) allocation of sufficient resources and realistic timelines for proper methodological development, rather than compressing complex scientific work into politically driven deadlines.

Establishing a unified methodology for evidence assessment will face challenges due to differing epistemological perspectives. A prior epistemological and normative alignment is required to ensure the creation of a robust, shared methodological guidance document. While redesigning the EU-HTA R is feasible, it necessitates a precise and coherent roadmap to circumvent current limitations. Despite these challenges, the EU-HTA R is expected to continue and gradually progress towards becoming an agency with a broader scope. This aligns with the vision of EU leaders to harmonize decision-making processes at the European level.

In synthesis, this manuscript has examined the EU-HTA R across three complementary dimensions (moral, epistemological, and ontological, as elaborated in Section 4.2). Part I demonstrated, through a systematic assessment of fourteen operational objectives, that the regulation faces significant implementation challenges: the scope of JCAs is restricted to clinical evidence stripped of scientific judgement and contextualization; guidance documents exhibit methodological deficiencies inconsistent with established HTA standards; resource requirements substantially exceed current allocations; and stakeholder engagement has been procedurally inadequate. Part II established that failure to meet these objectives carries consequences beyond the HTA domain, potentially eroding the epistemic authority of European institutions and setting precedents for substandard regulatory science. Part III revealed that the root cause of many implementation difficulties lies in unresolved epistemological divergences among Member States—divergences that span fundamental choices between Bayesian and frequentist inference, Fisher and Neyman–Pearson hypothesis testing frameworks, positivist and constructivist methodologies, and utilitarian and deontological ethical orientations.

The policy implications of these findings are substantial. First, the current trajectory of EU-HTA implementation risks producing JCA reports that Member States will find insufficient for their national decision-making, thereby failing to achieve the regulation’s primary objective of reducing duplication and improving efficiency. If national HTA bodies must conduct supplementary assessments to compensate for the limitations of JCA reports, the net effect may be an increase rather than a decrease in the overall burden of HTA across Europe. Second, the absence of an explicit epistemological consensus means that apparently technical disagreements—over acceptable evidence thresholds, the role of indirect comparisons, or the handling of uncertainty—are in fact unresolved philosophical disputes that cannot be settled through procedural compromise alone. Third, the credibility of the EU-HTA process as a model for evidence-based governance is at stake: if the first mandatory JCAs produce assessments of demonstrably lower quality than established national HTAs, this will not only undermine confidence in the EU-HTA R specifically but may also damage the broader project of European regulatory harmonization.

Looking ahead, three avenues for future action emerge from this analysis. The scheduled 2028 review of the EU-HTA R provides a critical window for corrective action; our proposed phased roadmap (Section 4.3)—moving from objective clarification through epistemological alignment to institutional redesign and operational guidance—offers a structured pathway for this revision. In the shorter term, upgrading the quality of guidance documents and templates through the safeguards outlined above could partially mitigate implementation risks without requiring legislative amendment. Finally, the broader academic and policy community should engage in developing the epistemological consensus that this analysis has shown to be absent—through dedicated working groups, peer-reviewed methodological publications, and structured dialogue between the distinct HTA traditions represented across Member States. The success of the EU-HTA R will ultimately depend not on the ambition of its legislative mandate but on the rigour, transparency, and intellectual honesty with which it is implemented.

Importantly, the challenge of achieving epistemic alignment across heterogeneous systems is not without precedent in healthcare. Recent empirical evidence demonstrates that standardized terminologies integrated into electronic health records have already been used to operationalize such alignment in clinical practice. For instance, structured approaches to harmonizing clinical data standards across diverse healthcare settings have shown that consensus on foundational concepts—such as what constitutes a valid clinical observation, how outcomes should be classified, and which measurement frameworks should be privileged—can be achieved through iterative, evidence-informed processes (PMID: 39381892; 39857934). These examples from clinical informatics and health data governance suggest that the epistemological alignment we advocate for HTA is not merely a theoretical aspiration but a pragmatically attainable objective, provided that the process is structured with sufficient rigour, transparency, and stakeholder engagement. The key lesson from these domains is that alignment need not imply uniformity: shared frameworks can accommodate context-specific applications while maintaining coherence at the methodological core.

## 4. Part III: A Common Epistemological Framework Is a Pre-Condition for Defining a Joint HTA Framework

The EU-HTA R objectives may be hindered by the differing epistemological frameworks of various MS HTA bodies. To establish a unified JCA methodology, representatives must first agree on the foundational principles of HTA. Without this alignment, creating a clear and consistent methodology remains impossible. Discussing methodology separately from its epistemological basis leads to disagreements and vague, non-prescriptive guidance documents.

In Part III, the manuscript proposes an epistemological framework to be considered for developing joint HTA regulation and JCA across EU Member States.

### 4.1. Philosophical Choices Drive EU-HTA Heterogeneity

While numerous studies on policy comparisons have examined the differences between various EU HTA evidence and value frameworks [59,60,61], insufficient attention has been given to understanding the root causes of this heterogeneity. The primary issue lies in comprehending the foundational differences between various MSs’ HTA processes, which originate from distinct philosophies concerning healthcare models and experimental assessments. The methodological framework of MSs HTA bodies is influenced by deliberate or inadvertent epistemological decisions. Prior to discussing the alignment of MSs behind a unified methodology for clinical assessment, it is crucial to understand the fundamental philosophical criteria that inform these methodological choices. This section reviews the elements of a philosophical framework that may serve this purpose. It will be of the utmost importance that MSs align on philosophical dimensions before considering developing joint methodological guidelines and appreciating historical decisions on health care organization and funding.

As this topic is very important for setting a sustainable joint methodological collaboration on JCA for HTA, we propose to describe briefly some important epistemological dimensions influencing the HTA decision analysis framework, because this information is not available in a single accessible consolidated document.

#### 4.1.1. Beveridge vs. Bismarck

The Beveridge model treats healthcare as a right for all citizens, regardless of income or employment status, providing universal access. It emphasizes equity and strives to ensure everyone has equal access to care. As it is managed by the state, there is a strong focus on cost control [26,27].

The Bismarck model uses mandatory health insurance funded by employer and employee contributions, providing health care coverage to employees. It may result in differences in coverage for low-income or unemployed individuals or those in rural areas. This model is considered efficient but also incurs higher costs [26,27].

The distinctions between Bismarck and Beveridge healthcare models are becoming blurred as countries integrate elements from both systems [62].

Both systems are evolving towards more inclusive and regulated healthcare delivery. However, the foundation continues to lead different visions and aims for both models.

#### 4.1.2. Utilitarian vs. Deontological Model

Accumulated regulations, laws, and practices aim to address the limitations within each system (Beveridge and Bismarck), thereby narrowing both. The underlying philosophy influences the perspective of the assessment culture. The deontological approach is often linked to the Bismarck countries model, whereas the utilitarian approach is frequently associated with the Beveridge countries model [63,64].

The deontological model emphasizes patient-centred rights and duties, focusing on autonomy, dignity, informed consent, and confidentiality. It considers absolute moral rules or duties. In contrast, the utilitarian model prioritizes societal benefits, aiming to maximize good for the majority by considering population-level outcomes and using cost-effectiveness for resource allocation [63].

#### 4.1.3. Ontic Component vs. Epistemic Component

How MS HTA bodies privilege the ontic or epistemic component in their HTA framework is also critical. The philosophical distinction between ontic (objective reality) and epistemic (knowledge-based reality) components of experiments impacts how evidence is apprehended by assessors conducting the experiment [65].

The ontic component evaluates whether the experiment aligns with the state of the art. The answer is binary: yes or no. If the experiment does not comply with these standards, it is rejected. This assessment is objective and undisputed, assuming there is consensus on what constitutes the state of the art [65]. However, understanding how the state of the art is defined in the EU-HTA R is not straightforward. It matches a regulatory question that is binary.

The epistemic component focuses on how we acquire and assess knowledge regarding health technologies. It involves the interpretation by the author of the experiment, based on his realistic assessment derived from the experiment. This is not a binary concept but rather a continuum. It addresses for example methodologies for handling incomplete or conflicting data and maintains inclusivity by incorporating diverse forms of evidence, including clinical trials, real-world data, and patient experiences [65]. It matches understanding what the potential benefit is and therefore the value of a health care intervention.

#### 4.1.4. Bayesian Statistical Model vs. Frequentist Model

Bayesian and frequentist statistics are two fundamental approaches to statistical inference, each with distinct philosophies and methodologies. Bayesian probability represents a degree of belief or uncertainty about an event or parameter while incorporating prior knowledge and updating beliefs based on new evidence. Frequentist probability is interpreted as the long-run frequency of an event occurring in repeated trials. It focuses on the properties of statistical procedures over hypothetical repeated sampling [66,67]. Intense epistolary debates animated the topic between Bayesians and frequentists but mostly among frequentists, Fisher on one side and Neyman & Pearson on the other side.

Although frequentist statistics dominate the field in clinical trial research, we see more slowly emerging Bayesian statistics-supported research in the field.

Bayesian statistics start with prior beliefs (prior distribution) about parameters, update these beliefs using observed data (likelihood) and produce a posterior distribution that combines prior and data. Frequentist statistics rely solely on observed data, use hypothesis testing and confidence intervals, and do not incorporate prior beliefs explicitly [67].

Bayesians explicitly incorporate prior distributions, which encode subjective or objective knowledge about parameters before observing data. This approach allows for direct probability statements about hypotheses, unlike frequentist methods [67]. It allows one to consider comprehensive information prior to the new experiment to answer a specific question.

While HAS and IQWIG favour frequentist statistics over Bayesian ones [68,69], most HTA driven by cost-effectiveness analysis favour Bayesian statistics in several situations for indirect treatment comparison [70,71,72]. Recently IQWIG suggested introducing Bayesian statistics in their next methodological guidelines [73].

#### 4.1.5. Fisher Perspective vs. Neyman & Pearson One

Fisher and Neyman–Pearson reject the use of priors, arguing that they introduce subjectivity and lack a clear frequentist interpretation. They considered the reliance on subjective priors, which they viewed as incompatible with scientific objectivity [74].

Fisher’s significance testing focuses on measuring evidence against a null hypothesis through *p*-values. A *p*-value quantifies the probability of observing data as extreme as (or more extreme than) the actual data, assuming the null hypothesis is true. Fisher viewed this as a tool for inductive inference, where the *p*-value helps assess whether data contradict the null hypothesis, without explicitly considering alternative hypotheses [75,76].

The Neyman–Pearson hypothesis testing formalizes decision-making between competing hypotheses (null vs. alternative). It emphasizes controlling Type I (false positive) and Type II (false negative) error rates via predefined significance levels (e.g., α = 0.05) and power analysis [76]. This framework treats testing as “inductive behavior,” where decisions (reject/accept) are guided by error probabilities rather than evidence quantification.

Both visions are driven by philosophical divergences. From a probability interpretation perspective, Fisher aligned with fiducial probability, while Neyman–Pearson adopted frequentist error rates [77]. From a purpose perspective, Fisher aimed to update scientific understanding through probabilistic evidence, whereas Neyman–Pearson prioritized actionable decisions.

Different HTA agencies adopt one or the other frequentist perspective; France aligns more with the Neyman and Pearson perspectives [68], the United Kingdom uses the Fisher method [78], while Germany oscillates between the two [69,79]. It is interesting to notice that IQWIG in Germany, in the draft of methodology version 8, states that they will increase the use of Bayesian statistics in the future [73].

It is important to acknowledge that formal hypothesis testing plays a limited direct role in most HTA systems. HTA is primarily concerned with the quantification of the added value of an intervention, and therefore the key questions centre on estimation—of treatment effects, uncertainty, and clinical relevance—rather than on binary accept/reject decisions derived from statistical significance testing. With the notable exceptions of France, which explicitly applies the Neyman–Pearson framework to determine whether a statistically significant difference exists, and Germany, where IQWiG employs confidence interval-based rules that operationally embed hypothesis testing logic, most national HTA bodies do not rely on formal statistical significance thresholds. Nevertheless, these philosophical distinctions remain relevant to the broader epistemological discussion because they shape how evidence certainty is interpreted, how *p*-values and confidence intervals are used as heuristics for evidence strength, and how the burden of proof is implicitly allocated between innovators and payers. Understanding these underpinnings is therefore essential for aligning a joint European methodology, even where formal testing is not the primary analytical tool.

#### 4.1.6. Positivist vs. Constructivist Approaches

The positivist approach in HTA involves evaluating information through clinical trials, cost-effectiveness analysis, and other quantitative methods [80]. It emphasizes methodological rigour to predict outcomes and guide healthcare decisions based on quantitative evidence-based models.

The constructivist approach in HTA incorporates subjective values like patient preferences, ethical considerations, and societal impacts into evaluations [80]. It emphasizes understanding diverse stakeholder perspectives to inform decision-making in a socially responsive manner using qualitative evidence.

The positivist paradigm emphasizes objectivity, prediction, and control using quantitative methods, while the constructivist paradigm values subjective realities, contextual understanding, and qualitative insights. Both paradigms have distinct strengths that can complement each other in fields like HTA. However, it remains difficult for qualitative methods to weigh in front of quantitative outcomes as considered too often as soft evidence [81].

#### 4.1.7. Evidence Hierarchy

Although RCTs and meta-analyses are widely recognized as the highest level of evidence, they often have high internal validity but low external validity. They may not predict adverse events in large populations or under different conditions of use, nor rare events [82]. Observational studies risk confounding and fishing analyses or data dredging bias [83]. New disruptive technologies may offer promising results with limited evidence, challenging a predefined evidence hierarchy framework. Balancing endpoints like improved efficacy and worse safety, quantitative and qualitative research, remains difficult. The established evidence hierarchy often confronts hard realities. The position of evidence within the hierarchy of various types of studies can change depending on the specific question being addressed [84].

#### 4.1.8. Separation vs. Integration of Evidence Assessment and Appraisal

Although it is debatable whether the decision to separate assessments from appraisal within the HTA organization is an epistemological decision or just an operational process for responsibility split, it remains an important question. While countries separating both processes may find an easy split between the EU-HTA role and the MS HTA bodies’ role, it may be more complex for countries managing both steps integrated.

The evidence assessment defines the process of collecting, reviewing, and synthesizing clinical and economic evidence to evaluate the safety, effectiveness, and cost-effectiveness of health technologies. It focuses on scientific evidence and objective analysis [85].

The appraisal defines the interpretation of assessment findings within the context of a specific healthcare environment, incorporating social value judgements, ethical considerations, and local priorities. It focuses on the contextualization of evidence to inform decision-making [85].

### 4.2. Norms in HTA Are Inevitable

HTA cannot be value-neutral due to its basis in moral, epistemological, and ontological commitments. Moral norms prioritize desirable health technology outcomes like patient autonomy or equity. Epistemological norms guide evidence collection, often preferring RCTs over real-world data. Ontological norms determine relevant effects, such as clinical outcomes versus quality of life [65].

It seems difficult to consider developing a joint evidence assessment process without alignment on the epistemological concepts that sustain the scope of assessment and the scientific evidence assessment method. This alignment is necessary to ensure guidance documents are developed in a consistent and meaningful way. Without agreement on such an epistemological platform, guidance developers will face the challenge of conciliating the irreconcilable.

To ensure the EU-HTA R aligns with guidance documents, methodologic choices, and templates it should follow a strict process where identification of a joint epistemological framework is a critical step.

### 4.3. Proposal to Address EU-HTA R Challenges and Align Objectives with Guidance Documents and Template

The preceding analysis (Section 4.1 and Section 4.2) has demonstrated that the heterogeneity of EU-HTA methodologies is not merely a technical divergence but is rooted in fundamental epistemological choices—spanning healthcare system philosophies (Beveridge vs. Bismarck), ethical frameworks (utilitarian vs. deontological), statistical paradigms (Bayesian vs. frequentist; Fisher vs. Neyman–Pearson), and the role of norms in evidence assessment. A central finding is that the current EU-HTA R guidance was developed without first establishing consensus on these foundational dimensions, which explains the resulting ambiguity and low prescriptive quality of the guidance documents. Building on this diagnosis, the following operational proposal outlines a logical sequence of steps designed to address these root causes: first establishing clear objectives, then achieving epistemological alignment, before proceeding to institutional design and, finally, the creation of operationally consistent guidance. This sequencing ensures that methodological choices are grounded in explicit and shared philosophical foundations rather than being imposed through procedural shortcuts.

A legitimate counter-argument is that methodological heterogeneity across Member States may itself represent a form of epistemic resilience rather than a failure requiring correction. Diversity of analytical approaches can provide multiple perspectives on evidence, enhance context-sensitivity, and guard against the systematic blind spots inherent in any single framework. We acknowledge the force of this argument and do not advocate for complete methodological uniformity. However, for a joint clinical assessment to produce outputs that are coherent, scientifically credible, and usable by all Member States, a baseline level of epistemological alignment is necessary. The goal should be understood as sufficient alignment for joint assessment—a shared understanding of foundational principles (what counts as evidence, how uncertainty is characterized, what role values play in assessment) that still permits Member States to apply context-specific frameworks at the appraisal stage. Drawing on a realist epistemological perspective, we recognize that multiple methodological frameworks may be utilized as appropriate to the research question at hand—a fit-for-purpose approach—without this implying indecision or rigidity. What is essential is that the choice of framework be explicit, justified, and mutually understood.

This raises the question of who should define the epistemic authority underpinning a joint HTA framework. We argue that epistemic authority in this context should not derive from institutional position alone—neither from the European Commission, nor from any single national HTA body, nor from industry stakeholders. Rather, it should emerge from a transparent, rigorous, and consensual process involving all Member States with substantive input from the broader scientific community. The legitimacy of any shared epistemological framework depends on its being developed through procedures that themselves embody the epistemic virtues of transparency, systematic evidence review, and openness to critique. The proposed steps below are designed to instantiate this principle.

- Step 1: To define meaningful, clear, achievable and transparent strategic objectives for the EU-HTA R. This is the primary goal of the EU-HTA R.
- Step 2: To assess the feasibility and scope of an EU-HTA process. This will require mapping the underlying epistemic foundation of each individual MS HTA framework. Then agree on a common epistemological model. Then agree on norms. HTA relies on a normative tri-dimensional process. Without a common epistemological normative model, it is not possible to consider a robust shared framework.
- Step 3: To reassess the best option to achieve the set objective, through a secretariat or an agency for example. Preference for an agency with a single framework seems to be slowly emerging even though the impact study performed in 2018 concluded that the preference for a secretariat [86].
- Step 4: To develop the operational objectives that contribute to achieving the strategic objectives.
- Step 5: To develop the operational framework that is consistent with the strategic and operational objectives as well as the commonly agreed epistemological framework.
- Step 6: To perform an impact study that was not done for EU-HTA R a priori.
- Step 7: To consolidate the previous workflow and develop a consistent law/regulation.
- Step 8: Once the epistemological framework is aligned and the law is voted on, the development of methodological guidance should naturally flow as derived from the epistemological framework agreed upon among MSs.

The development should be done by people skilled in the art and following well-established processes including systematic literature review, optimal method recommendation, expert and public consultation, decision and publication. Authors should be identifiable and accountable, and all process steps should be made transparent to third parties. It may be that a common framework does not emerge. In that case we may end up with two frameworks and have two different assessments, or the EU Commission may invite the top EU experts in the field to build a consensus on a single framework. While consensus is feasible to reach on medical practice, it is more complex to achieve on epistemological issues.

## Data Availability

No new data were created or analyzed in this study. Data sharing is not applicable to this article.

## Supplementary Materials

The following supporting information can be downloaded at: https://www.mdpi.com/article/10.3390/jmahp14030045/s1. File S1: OECD DAC Evaluation Framework Applied to the EU Health Technology Assessment Regulation (EU-HTA R).

## Abbreviations

The following abbreviations are used in this manuscript:

ATMPs	Advanced Therapeutic Medicinal Products
CMA	Critical Medicine Act
EBM	Evidence-Based Medicine
EU	European Union
EU-HTA	European Union’s Health Technology Assessment
EU-HTA R	European Union’s Health Technology Assessment Regulation
GDP	Gross Domestic Product
HAS	Haute Autorité de Santé
HTA	Health Technology Assessment
HTDs	Health Technology Developers
IQWIG	Institute for Quality and Efficiency in Health Care
JCAs	Joint Clinical Assessments
MS	Member State
PICO	Population, Intervention, Comparator, Outcome
RCTs	Randomized Clinical Trials
