# Supplementary material for: The European Union’s Health Technology Assessment Regulation (EU-HTA R) Will Prosper Despite Major Setbacks
Source: J Mark Access Health Policy. 2026 Aug 3;14(3):45. doi: 10.3390/jmahp14030045 (PMC13510476; doi:10.3390/jmahp14030045)
Supplement: Supplementary file 1 [file jmahp-14-00045-s001.zip › jmahp-3807882-supplementary.pdf]

File S1: OECD DAC Evaluation Framework Applied to the EU Health Technology Assessment Regulation (EU-HTA R)

1. What is the OECD DAC Evaluation Framework?

The OECD Development Assistance Committee (DAC) Network on Development Evaluation developed a set of evaluation criteria first formalised in 1991 and comprehensively revised in 2019. The 2019 revision aimed for clearer definitions, increased focus on equity, and a stronger emphasis on sustainability of benefits rather than just financial viability. Originally designed to assess the quality and performance of international development programmes and aid interventions, the DAC criteria have since been adopted far beyond their original context and are now widely applied to public policy evaluation, regulatory impact assessment, health system reform, and institutional governance reviews. The framework is endorsed by the United Nations Evaluation Group (UNEG) and constitutes the global reference standard for structured programme evaluation.

The six criteria — Relevance, Coherence, Effectiveness, Efficiency, Impact, and Sustainability — provide a structured, multi-dimensional lens through which any policy intervention can be assessed in a systematic and reproducible manner. Each criterion addresses a distinct evaluative question, together covering the full lifecycle of a policy from its design rationale through to its long-term effects. The framework is deliberately criterion-based rather than metric-based, allowing expert judgement to be rendered explicit, transparent, and contestable — precisely the properties required for a reproducible evaluation of a regulatory instrument such as the EU-HTA R.

In this appendix, the six DAC criteria are applied at the level of individual objectives stated in the EU-HTA Regulation. Each objective is scored on a five-point Likert scale (described in Section 3), and the rationale for each score is grounded in the evidence and arguments developed in the main manuscript. This framework is intended to serve as a reproducible instrument: independent evaluators revisiting the same evidence base should be able to apply identical criteria definitions and arrive at comparable scores, or explicitly identify the grounds for divergence.

2. Definition of Each DAC Criterion and Its Application to EU-HTA R

Relevance. In standard DAC usage, relevance asks whether the intervention addresses genuine needs and aligns with the priorities of intended beneficiaries and partner countries. It evaluates the fit between the stated objectives and the real-world problem the policy is designed to solve.

In the EU-HTA R context, relevance assesses whether each stated objective genuinely corresponds to a documented gap or need in the European health technology assessment landscape — specifically, disparities in patient access to innovation, inefficiencies from duplicated national HTA processes, and the absence of a shared methodological framework. An objective is scored as highly relevant when it

addresses a well-documented structural problem; lower scores reflect objectives whose connection to demonstrated need is ambiguous or where the policy instrument is unlikely to be the appropriate lever.

Coherence. DAC coherence examines the compatibility of the intervention with other policies, both internal (within the same institutional framework) and external (with policies of partner entities). It identifies contradictions and synergies.

Applied to EU-HTA R, coherence evaluates whether each objective is mutually consistent with other objectives within the Regulation, with existing EU law (notably subsidiarity in healthcare under Article 168 TFEU), with Member State HTA traditions, and with parallel EU legislative initiatives (Critical Medicines Act, pharmaceutical legislation revision). Low coherence scores are assigned where structural contradictions are identified — such as the simultaneous pursuit of harmonisation and respect for subsidiarity, or the expectation of high scientific quality without the institutional architecture (authorship, peer review, SLR) that quality requires.

Effectiveness. Effectiveness in DAC terms asks whether the intervention achieves, or is likely to achieve, its stated objectives. It is the most direct evaluative question: does the policy do what it says it will do?

In this application, effectiveness is the central criterion. It is scored according to the probability that each stated objective will be achieved under current implementation conditions — drawing on the analysis of guidance document quality, resource constraints, structural barriers, and procedural deficiencies documented in the main manuscript. A score of 1 reflects the manuscript's conclusion that an objective is very unlikely to be achieved; a score of 5 would indicate that enabling conditions are fully in place. Most objectives in the present analysis receive scores of 1 or 2, consistent with the manuscript's overall thesis.

Efficiency. DAC efficiency examines whether the outputs and outcomes of an intervention are commensurate with the resources invested — financial, human, institutional, and temporal. It distinguishes between interventions that deliver value for money and those that generate disproportionate costs relative to benefits.

In the EU-HTA R context, efficiency is assessed by comparing the administrative and scientific burden placed on Member States, HTA bodies, and Health Technology Developers against the expected benefit of the JCA process. Empirical evidence of inefficiency is strong: Sweden's estimate of 17 additional FTEs, AIFA's resource concerns, the 90-day submission window that generates uncertainty without commensurate clarity, and the fragmented PICO structure that increases — rather than consolidates — reading burden. An objective scores low on efficiency when the costs of compliance demonstrably exceed the value delivered.

Impact. Impact in DAC methodology refers to the significant and lasting changes — positive or negative, intended or unintended — that an intervention produces in the broader system it operates within. It looks beyond immediate outputs to second-order and systemic effects.

For EU-HTA R, impact evaluates whether each objective, if achieved, would produce a meaningful change in the European pharmaceutical landscape: in patient access, in HTA quality, in market functioning, or in scientific standards. Many objectives receive low impact scores not because they are unimportant in design, but because the Regulation's restricted scope (clinical assessment only, no appraisal, no pricing)

limits the causal chain between JCA outputs and real-world patient or system outcomes. Where the Regulation is structurally incapable of reaching the causal lever required to produce the intended change, impact is scored accordingly.

Sustainability. DAC sustainability asks whether the benefits produced by an intervention are likely to continue after the intervention ends, or — in the case of an ongoing policy — whether the institutional, financial, and political conditions exist to maintain and develop the intervention over time.

Applied to EU-HTA R, sustainability evaluates the durability of any gains attributable to each objective, as well as the long-term institutional viability of the JCA process itself. Low sustainability scores reflect situations where the scientific credibility deficits identified in the manuscript — particularly the absence of peer-reviewed, author-attributed guidance — risk delegitimising the process before it matures. Higher sustainability scores are assigned where objectives are institutionally anchored (e.g., administrative standards) or where the EU cooperation structure provides a sufficient organisational foundation for improvement over time.

3. Scoring Scale — Five-Point Likert Scale with Anchored Extremes

Each objective is scored on a five-point Likert-type scale for each of the six DAC criteria. The scale is anchored at both extremes with explicit interpretive definitions to ensure inter-rater reproducibility. The midpoint (3) denotes genuine uncertainty or mixed evidence rather than an absence of judgement. Scores are not averaged across criteria; the multi-dimensional profile is intentionally preserved to allow readers to identify which specific evaluative dimension accounts for a given objective's difficulty.

Score	Meaning (colour-coded)	Interpretive anchor
**1**	1 — Objective very unlikely to be achieved / performance very poor	Structural or design barriers make achievement implausible under current conditions
**2**	2 — Objective unlikely to be achieved / performance poor	Significant obstacles identified; partial achievement at best without major reform
**3**	3 — Achievement uncertain / performance moderate	Mixed evidence; outcome depends on future governance decisions or additional resources
**4**	4 — Objective likely to be achieved / performance good	Conditions largely favourable; minor gaps remain
**5**	5 — Objective very likely to be achieved / performance strong	All enabling conditions present; achievement expected under current trajectory

4. Evaluation Matrix

The table below presents the full objective-level evaluation. Each row corresponds to one stated objective of the EU-HTA R, categorised by tier (Strategic, Operational, Specific). Scores on all six DAC criteria are provided, with colour coding corresponding to the legend above. The rationale column summarises the evidentiary basis for each score as developed in the main manuscript.

Category	Objective	Relevance	Coherence	Effectiveness	Efficiency	Impact	Sustainability	Key Rationale (based on manuscript findings)
**STRATEGIC OBJECTIVES**								
Strategic	High level of health protection for patients and users	5	3	1	2	2	3	Access disparities driven by GDP/affordability differences, not HTA. EU-HTA R has no pricing or procurement mandate; structural cause unaddressed.
Strategic	Smooth functioning of the internal market for medicinal products	5	2	1	2	1	2	Subsidiarity in healthcare, divergent national frameworks, and restricted clinical-only scope make contribution to single market functioning negligible.
**OPERATIONAL OBJECTIVES**								
Operational	Improve availability of innovative technologies for EU patients	5	3	1	2	1	2	Overlaps with strategic objective 1. No mechanism within EU-HTA R to address affordability or procurement; access disparities persist.
Operational	Harmonize HTA criteria and	5	2	1	2	2	1	Guidance vague and subject to divergent MS interpretation; 'due

Category	Objective	Relevance	Coherence	Effectiveness	Efficiency	Impact	Sustainability	Key Rationale (based on manuscript findings)
	increase transparency							consideration' undefined; no binding obligation on MS to follow JCA conclusions.
Operational	Ensure efficient use of resources and strengthen quality of HTA	5	2	1	1	2	1	Sweden estimates +17 FTEs; AIFA resource concerns documented. Guidance developed without SLR, authorship, or external review — constitutes eminence-based medicine.
Operational	Improve business predictability for health technology developers (HTD)	5	2	1	2	1	2	90-day timeline insufficient; no applicant guidance on methodological requirements; scope uncertainty high; unpredictable MS interpretation of JCA reports likely worsens predictability.
**SPECIFIC OBJECTIVES**								
Specific	Comply with the state of the art of medical sciences / EBM	5	2	1	2	1	1	Guidance lacks systematic literature review, identified authorship, and external peer review. Reflects 'eminence-based medicine' (Isaacs & Fitzgerald, BMJ 1999) — the lowest EBM level.
Specific	Timely and up-to-date assessments	4	2	2	2	2	3	JCA reports available 180–330 days post-EMA marketing authorization. Stop-the-clock integration

Category	Objective	Relevance	Coherence	Effectiveness	Efficiency	Impact	Sustainability	Key Rationale (based on manuscript findings)
								adds unpredictability. Accelerated pathway raises feasibility concerns for assessors.
Specific	Inclusive stakeholder and expert involvement	5	2	1	2	2	2	Guidance developed without genuine public consultation; HTDs and broad expert communities excluded from development process; stakeholder diversity insufficient.
Specific	Adhere to highest standards of quality, impartiality, transparency, and independence	5	2	1	2	1	2	Arbitrary tool selection (ROBINS-I, ICEMAN); negative a priori on non-RCT evidence; guidance reflects viewpoints of specific HTA bodies lacking balanced methodological perspective.
Specific	Minimize duplicate efforts for MSs and HTD	5	2	1	1	1	2	New obligations added without removing national ones. Fragmented PICO structure across JCA report increases reading burden. Country-specific submissions required in parallel — duplication not reduced.
Specific	Adhere to the highest administrative standard	4	3	3	3	3	3	Achievable over time as EU institutional capacity matures. Current gaps reflect early-stage voluntary MS coordination rather than structural failure. Guidance

Category	Objective	Relevance	Coherence	Effectiveness	Efficiency	Impact	Sustainability	Key Rationale (based on manuscript findings)
								documents explicitly disclaim EU Commission status.
Specific	Adapt to specific needs of emerging health technologies (vaccines, orphan drugs, ATMPs)	**5**	**2**	**1**	**2**	**1**	**2**	Templates and guidance show no adaptation to emerging technologies. Directly contrary to regulatory text requirements. No effort documented to ensure compliance in this area.
Specific	Focus primarily on RCT while considering alternative evidence	**4**	**2**	1	**2**	1	**2**	Alternative designs acknowledged but carry negligible evidentiary weight. Negative a priori on observational studies and SATs discourages their submission. Stated objective unlikely to be met in practice.
Specific	Reduce the cost of HTA submissions for HTDs	**4**	**2**	**1**	**1**	**1**	**2**	Country-specific submissions required in addition to JCA submission. JCA does not replace national dossiers. Overhead costs likely to increase rather than decrease for HTDs.
Specific	Establish sustainable EU HTA cooperation	**4**	**3**	**3**	**3**	**3**	**3**	Structural cooperation feasible as EU coordination mechanisms mature. Scientific credibility gaps and unresolved epistemological divergences may

Category	Objective	Relevance	Coherence	Effectiveness	Efficiency	Impact	Sustainability	Key Rationale (based on manuscript findings)
								undermine long-term institutional sustainability.
**Scoring legend:** 1 = Very Unlikely / Very Poor | 2 = Unlikely / Poor | 3 = Uncertain / Moderate | 4 = Likely / Good | 5 = Very Likely / Strong								

5. Score Distribution by DAC Dimension

The table below reports, for each of the six DAC criteria, the number of objectives receiving each score (1–5), the total number of objectives evaluated (n = 16), and the modal score and distributional pattern. A narrative interpretation follows.

DAC Dimension	5	4	3	2	1	Total	Modal score & pattern
**Relevance**	11	5	—	—	—	16	Modal: 5 | Narrow range (4–5)
**Coherence**	—	—	4	12	—	16	Modal: 2 | Narrow range (2–3)
**Effectiveness**	—	—	2	1	13	16	Modal: 1 | Spread (1–3)
**Efficiency**	—	—	2	11	3	16	Modal: 2 | Spread (1–3)
**Impact**	—	—	2	5	9	16	Modal: 1 | Spread (1–3)
**Sustainability**	—	—	4	9	3	16	Modal: 2 | Spread (1–3)

Interpretation by Dimension

Relevance. All 16 objectives score 4 or 5, with 11 reaching the maximum. The regulation addresses well-documented structural gaps — patient access disparities, duplicated national HTA work, and methodological fragmentation — and its stated goals are unambiguously

pertinent to real EU policy needs. Relevance is the one dimension where the EU-HTA R is unimpeachable: the right problems are identified, even if the instrument cannot solve them.

Coherence. All 16 objectives score 2 or 3; no objective achieves a score above 3. The dominant score of 2 (12/16) reflects deep structural contradictions: the simultaneous pursuit of harmonisation and subsidiarity in healthcare, the expectation of scientific quality without the institutional apparatus (peer review, authorship, SLR) to produce it, and the restricted clinical-only scope that leaves appraisal — the decisive decision step — entirely at Member State level. The four objectives scoring 3 are those where voluntary EU cooperation provides at least partial institutional support.

Effectiveness. Thirteen of 16 objectives score 1 — the most concentrated failure across the entire framework. Only two objectives (administrative standards and sustainable cooperation) reach a moderate score of 3, and a single objective (timely assessments) scores 2. This near-uniform floor reflects the manuscript's central thesis: the regulation was designed without prior epistemological alignment among Member States, producing guidance documents too weak to drive coherent implementation. Effectiveness is the most critical DAC dimension and its distribution is the starkest finding of this evaluation.

Efficiency. Eleven objectives score 2 and three score 1, with only two reaching 3. The cost-benefit balance is consistently unfavourable: Sweden's estimate of 17 additional FTEs, Italy's resource concerns, the 90-day submission window, and the fragmented PICO structure all document a process that adds administrative and scientific burden without commensurate reduction in national workload. The three objectives scoring 1 — efficient resource use, minimising duplication, and reducing HTD submission costs — are precisely those the regulation most explicitly promised to deliver.

Impact. Nine objectives score 1 and five score 2; only two reach 3. The near-absence of positive impact potential reflects a structural ceiling inherent to the regulation's design: by restricting scope to decontextualised clinical assessment and excluding scientific judgement, appraisal, and pricing, the JCA process is causally disconnected from the outcomes it is intended to influence — patient access, market functioning, and HTA quality. Even if the JCA process were perfectly executed, its architectural limitations would prevent meaningful systemic change.

Sustainability. Nine objectives score 2 and four score 3; three objectives score 1. Sustainability is marginally stronger than effectiveness or impact, reflecting the institutional durability of EU cooperation structures. However, the guidance quality deficits identified in the manuscript — absence of authorship, SLR, and peer review — risk delegitimising the scientific basis of JCA outputs before the process matures. The four objectives scoring 3 (health protection, timely assessments, administrative standards, sustainable cooperation) are those anchored in EU institutional processes rather than dependent on methodological credibility.

Overall Assessment

Taken together, the score distributions present a coherent and consistent picture. The EU-HTA R scores uniformly high on Relevance — the policy rationale is sound — but collapses across the five implementation dimensions. Coherence and Sustainability are constrained to scores of 2–3, reflecting structural contradictions and fragile institutional foundations. Effectiveness, Efficiency, and Impact concentrate at scores 1–2, documenting a systematic gap between legislative ambition and operational capacity. The two objectives that consistently perform better across all dimensions (adherence to administrative standards and establishment of sustainable EU cooperation) share a common feature: they depend on institutional processes and political will rather than on the scientific and methodological quality of the guidance documents — the core weakness identified throughout this analysis. This pattern supports the manuscript's conclusion that regulatory design revision and comprehensive guidance reform, not incremental adjustment, are required for the EU-HTA R to deliver on its stated objectives.

Abbreviations: ATMP = Advanced Therapy Medicinal Products; DAC = Development Assistance Committee (OECD); EBM = Evidence-Based Medicine; FTE = Full-Time Equivalent; HTD = Health Technology Developer; JCA = Joint Clinical Assessment; MS = Member State; OECD = Organisation for Economic Co-operation and Development; PICO = Population, Intervention, Comparator, Outcome; RCT = Randomised Controlled Trial; SLR = Systematic Literature Review; TFEU = Treaty on the Functioning of the European Union.
